# The Development and Psychometric Validation of a Comprehensive Measure Assessing Fear of Incompetence among Adults Who Have a Family Member with Dementia

**DOI:** 10.1155/2020/1910252

**Published:** 2020-01-28

**Authors:** Ashley E. Thompson, Anca M. Miron, Jonathan M. Rogers, Rudy Rice

**Affiliations:** ^1^Department of Psychology at the University of Minnesota Duluth, Duluth, MN, USA; ^2^Department of Psychology at the University of Wisconsin Oshkosh, Oshkosh, WI, USA

## Abstract

Because the interpersonal skills of individuals with dementia often decline, family members may question their own ability to interact meaningfully. These family members may experience fear of incompetence (i.e., fear of being unable to relate in a meaningful way or take care of a close family member with dementia). Thus, the goal of this research was to develop, refine, and psychometrically validate a scale (Fear of Incompetence—Dementia Scale; FOI-D) assessing fear of incompetence in the context of relationships with a close family member diagnosed with dementia. Three online studies were conducted to accomplish the primary objective. In Study One, the factor structure of the FOI-D was assessed by conducting an exploratory factor analysis using data from 710 adults who indicated having a close living family member who had been diagnosed with dementia. In Study Two, the factor structure was validated via a confirmatory factor analysis and the psychometric properties were established using data from 636 adults who had a family member with dementia. Finally, Study Three determined the temporal consistency of the scale by retesting 58 participants from Study Two. The results from Study One indicated that the FOI-D Scale accounted for 51.75% of the variance and was comprised of three subscales: the Interaction Concerns subscale, the Caregiving Concerns subscale, and the Knowledge Concerns subscale. In Study Two, the three-factor structure was supported, resulting in a 58-item scale. Investigation of the psychometric properties demonstrated the FOI-D to be reliable and valid. In Study Three, the FOI-D Scale demonstrated excellent temporal consistency. This research provides future investigators, educators, and practitioners with an adaptable comprehensive tool assessing fear of incompetence in a variety of settings.

## 1. Introduction

Dementia is a neurodegenerative disorder that causes significant cognitive decline, thus negatively impacting quality of life of those affected by this disease [1]. The effects on cognition include deficits in problem solving skills, episodic memory, concentration, thinking, and interpersonal skills. As these symptoms progress, they create increasing burden on the family members of individuals with dementia [2–4]. In fact, the negative changes in adults with dementia often challenge their family member's ability to use existing knowledge of their loved one's preferences, values, abilities, and shared history, which leads to fear in their interactions with the family member with dementia [5].

In our current research, we borrowed a concept from social psychological literature—*fear of incompetence* [6, 7]—to refer to this type of relational fear. Wicklund and Scheuer [7] defined fear of incompetence as the fear of not performing at a level expected by the individual or society. In the context of relationships with a family member with dementia, we define fear of incompetence as *the fear of being unable to relate in a meaningful way, communicate, or take care of a close family member diagnosed with dementia*. In interpersonal relationships, this fear can lead to avoidance of face-to-face interactions [6, 7] and likely a host of other negative relationship outcomes. Thus, the goal of the current program of research was to construct and empirically validate a psychometric scale assessing fear of incompetence in the context of relationships with a close family member who had been diagnosed with dementia.

## 2. Fear of Incompetence: Conceptual Specification and Distinctions

Although research indicates that individuals acquire new domains of competence when a close family member develops dementia (e.g., interactional, communication; [8]), little research has been conducted into people's concerns with these competencies (i.e., their fear of incompetence). Using a qualitative approach, Miron et al. [9] found that young adults experienced four different types of concerns in their relationship with their grandparent with dementia: those related to the inability to maintain a meaningful relationship, lacking dementia interaction skills/knowledge, effects on a third person, and changes in the person with dementia. Moreover, fear of interactional incompetence led young adults to avoid face-to-face interactions with their grandparent/great-grandparent with dementia and instead to use an older family member as an interaction buffer by having that family member engage in face-to-face interactions with the relative with dementia, while they listened to the conversation in the background [9].

In the development of the current scale, we focused on measuring the second type of concerns (interactional) from Miron et al. [9] study, which encompasses concerns about lacking skills and knowledge such as perspective-taking and self-regulation skills, knowledge about dementia, as well as caregiving skills and knowledge. These interaction skills are important in sustaining meaningful relationships with close others with dementia [10, 11]. Indeed, caring for and caring about people with dementia require specific communication and interaction skills [5]. Eggenberger et al. [5] reviewed several communication skills training strategies in dementia care that focus on different interaction domains: verbal skills, nonverbal, and emotional skills (e.g., using perspective-taking to recognize unusual communicative attempts), behavioral management skills (e.g., using distraction to reduce aggressive behavior), instrumental skills (e.g., using tools such as memory aids), and theoretical knowledge about dementia and communication/interaction strategies. When developing our scale items, we sampled from these interaction domains with the goal of assessing fear of incompetence across various interaction and communication skills.

It is imperative to take into account these interaction/communication domains because a breakdown in any of these areas can have negative consequences for the wellbeing of all interaction participants, including increased feelings of burden, stress, and anxiety in family members [11]. Not knowing how to interact can instigate strong anxiety [12], which in turn can lead to avoidance behaviors [13]. Social and psychological resources can be employed to reduce avoidance behaviors in family members of those with dementia. One type of psychosocial intervention, for instance, involves holding individual and family counseling sessions with the goal of improving social support for the caregiver and helping the family understand the nature of dementia and the difficulties it presents [14, 15].

Moreover, comprehensive training programs have been designed specifically with the goal of improving interaction skills for dementia caregivers and family members. For instance, Ripich et al. [16] found that caregivers can be trained to structure questions that facilitate more successful communication exchanges with their family members with dementia. Shulman and Mandel [17] designed a series of workshops for family and friends of residents aimed at informing them of the nature of communication, how it is affected by the aging process, the psychological and neurological nature of communication impairments, and how to manage situations in which communication breaks down. These workshops resulted in increased understanding, increased satisfaction with the visits, and improved skills in using communication-facilitating techniques. What all of these interventions have in common is providing caregivers and family members of persons living with dementia with resources to more successfully interact and take care of their family members with dementia. Face-to-face interactions, as opposed to more passive ways of interacting such as the use of person buffers or the use of mediated communication channels (email or phone calls), allow for more meaningful interaction and relationship experiences [9, 18, 19]. Moreover, caregivers may often avoid face-to-face, person-focused, interactions with their loved one with dementia by replacing them with task-oriented interactions focusing on getting caregiving tasks accomplished [20]. This leaves the person with dementia unacknowledged and ignored. It is thus important to systematically measure fear of interactional incompetence to capture this interpersonal phenomenon in dementia relationships.

## 3. The Current Program of Research

The primary objective of the current research was to develop and refine a scale measuring adults' fear of incompetence related to interacting with a family member diagnosed with dementia, assesses its factor structure, and establish its psychometric properties. In Study One, items were selected, developed, piloted, and exposed to an exploratory factor analysis (EFA). In Study Two, a confirmatory factor analysis (CFA) was used to validate the factor structure and establish the psychometric properties of the scale. Finally, test–retest reliability was assessed in Study Three.

## 4. Study One: Item Development and Factor Structure Assessment

As a first step in the development of the scale, a comprehensive list of fears and concerns was created. After doing so, data collected from these items were subjected to an EFA and the internal consistency of the scale was evaluated.

### 4.1. Method

#### 4.1.1. Participants

Adults (18 years and older) residing in North America who indicated having a close living family member (self-defined by participants) who had been diagnosed with dementia participated in the study via an advertisement placed on Mechanical Turk® (MTurk®). MTurk® is a widely used online marketplace through which workers can complete a variety of tasks in exchange for a nominal fee [21]. Research suggests that the data provided by MTurk® is more representative than samples surveyed using traditional recruitment methods, including undergraduate convenience samples [21, 22]. Although 800 adults met eligibility requirements and agreed to participate in the study, 90 were removed from the data file due to attrition or validity concerns (e.g., failing to complete the survey in its entirety, responding incorrectly to one of four validity check items). Thus, our final sample comprised of 710 adults (210 men and 500 women) who were predominantly Caucasian (77%), with a mean age of 35.85 years (*SD* = 11.45, range = 18–75 years). The majority of participants characterized their family member as their “grandparent” (58%), with the remaining participants indicating that their family member was their “parent” (19%), “aunt/uncle” (12%), “great-grandparent” (2%), or “other” (9%). On average, participants reported knowing their family member for approximately 33.38 years (in most cases their whole life,* SD* = 12.05, range = 1–69 years) and that their family member was diagnosed with dementia an average of 4.18 years ago (*SD* = 3.87, range = 3 months–30 years).

#### 4.1.2. Measures

*Demographic Questionnaire. *Participants reported on their gender, age, ethnicity, employment status, relationship with their family member with dementia, and the demographic characteristics of their family member (e.g., their relationship with the participant, the length of their relationship).

*The Fear of Incompetence Scale—Dementia (FOI-D Scale). *Eighty items were included in the initial version of the FOI-D Scale. These items were drawn in part from related measures (e.g., [23]) and via extended qualitative pilot focus groups (for more information, see [9] and from an original study measuring fear of incompetence. Following the initial item development, a second round of pilot testing was conducted to ensure clarity and conciseness in wording and to establish content validity (i.e., that the scale provides adequate coverage of the subject being studied). To do so, 15 adults with family members with dementia (10 women, 5 men, obtained through word of mouth and snowball sampling) were administered the FOI Scale and were then interviewed after completion. The interview protocol included items assessing clarity of scale instructions, their experience with the scale items, the utility of the response scale, items that may be missing, etc. Based on their feedback, instructions were modified and seven additional items were included in the scale, resulting in a final scale of 87 items.

*Procedure. *After receiving ethics approval from our institutions' IRBs, participants were recruited via MTurk® via advertisements indicating that participants would be asked to complete a brief and anonymous online survey on “emotions experienced during interpersonal interactions.” The decision to withhold the purpose of the study was to ensure that participants were not fabricating a relationship with a family member with dementia in order to participate (and receive compensation). All interested participants were instructed to click the link to the online survey hosted on a secure web server. The first survey participants received was an eligibility measure that included seven items, only one of which was used to establish eligibility (“Do you have a close living relative or family member who has been diagnosed with dementia?”). The reason for including the additional screener items (i.e., “Approximately how many hours a day do you spend interacting with others?”) was to disguise the purpose of the study in order to prevent participants from responding in fraudulent ways (approximately 23% of interested participants met the criteria necessary for participating). Participants meeting the criteria (having a close living family member with dementia) were then directed to a consent form containing information about the study. Those not meeting the criteria received a message describing our appreciation for their interest, but that they were not eligible for the study at this time. After providing consent, eligible participants completed the survey, which took approximately 45 minutes and were compensated by receiving $1.00.

### 4.2. Results and Discussion

Data were cleaned according to procedures outlined by Tabachnick and Fidell [24]. After data cleaning, the multidimensional nature of the FOI-D Sale was assessed using a maximum likelihood EFA with a Promax rotation via SPSS Statistics software (version 25). A maximum likelihood EFA was selected because of the theoretical nature of the scale [25] and a promax rotation was determined appropriate in order to account for the potential relatedness between factors [26]. The results from the KMO test (0.98) and Bartlett's test of sphericity (*χ^2^* [3741] = 41945.30, *p* < .001), revealed that the sampling adequacy and intercorrelations between items were appropriate for an EFA.

After determining that an EFA was appropriate, the results from the EFA revealed nine factors with eigenvalues greater than one but the scree plot and the parallel analysis indicated that a three-factor solution was best. In particular, the scree plot (graphing the eigenvalues for each factor) depicted an elbow at the third factor. In addition, using a sample size of 710 and 87 total items, the parallel analysis randomly created eigenvalues that were compared with sample eigenvalues obtained from the EFA. Only three eigenvalues from the EFA exceed those produced by the parallel analysis.

Thus, a second maximum likelihood EFA with a promax rotation that restricted the model to three factors was conducted. Scale items were retained if they had a factor loading of .40 or above on one of the factors [27], but no cross loadings (i.e., greater than .40 on two or more factors). A total of 20 items failed to load on any of the three factors and three items loaded on more than one factor. However, based on the researchers' discretion, one item that failed to load on any of the three factors (“I will not know how to console a third person in the interaction [e.g., sibling, parent, friend] if my relative with dementia is having a bad day”) was retained. This item was a common theme during pilot work and was considered an important concern. Thus, the 22 items that cross-loaded or failed to load were removed and a final EFA was conducted with the remaining items (see Table 1 for all omitted items).

Using the final 65 items, the final maximum likelihood EFA with a promax rotation was conducted. Again, the KMO test result for the final EFA was 0.98 and the Bartlett's test of sphericity was significant, *χ^2^* (2080) = 30149.64, *p* < .001. The three resulting factors were then named based on the items loading in each factor (see Table 2 for final factor loadings and communalities). The first factor, *Interaction Concerns* (accounting for 41.74% of the variance), was comprised of 17 items related to fears about one's competency in effectively relating and interacting with their family member. The second factor, *Caregiving Concerns* (accounting for 5.44% of the variance), consisted of 19 items related to fears/concerns associated with ensuring comfort, detecting the needs of, and providing care for one's family member. Finally, the third factor *Knowledge Concerns* (accounting for 4.57% of the variance), was comprised of 29 items related to fears/concerns stemming from ignorance or a lack of information about how to understand/communicate with one's family member. The internal consistency for each factor was high, as evidenced by their Cronbach alphas: Interaction Concerns = 0.97, Caregiving Concerns = 0.94, and Knowledge Concerns = 0.93 (see Table 2 for the subscale items). In sum, the results from Study One indicate that fear of incompetence is multifaceted and comprised of concerns related to interacting, caregiving, and knowledge.

## 5. Study Two: Scale Refinement and Validation

In Study One, the FOI-D Scale was developed and its three-factor structure was identified. This factor structure was validated using CFA performed on an independent sample in Study Two. The convergent, discriminant, and concurrent validity of the final scale was also assessed in Study Two.

### 5.1. Convergent Validity

Convergent validity was examined by assessing relationships between knowledge of dementia, attitudes toward dementia, caregiving burden, and the FOI-D Scale. Attitudes toward people with dementia have been documented to relate to feelings of discomfort and/or lack of confidence when interacting with a person with dementia (e.g., [28])—a conceptual dimension attitudes and fear of incompetence share. Caregiver burden is also likely conceptually related to fear of incompetence because of evidence indicating considerable anxiety, stress, and care burden in both family members and caregivers of persons with dementia (e.g., [29]). Although the current research did not focus exclusively on primary caregivers, it did focus on the fear of incompetence experienced by family members of individuals with dementia, who likely have extensive involvement in caregiving responsibilities and experience burnout [30, 31]. Finally, because fear of incompetence may manifest itself as fear of not knowing how to interact with a close family member with dementia due to the lack of relevant knowledge about dementia symptoms [9], we also assessed knowledge of dementia as a related but distinct construct. Indeed, in a study of assisted living communities, dementia residents who received care from nurses who had greater knowledge of caregiving responses received better and more regular care (i.e., higher approach behaviors) than did those who were assigned to less knowledgeable nurses. Thus, we predicted that dementia knowledge, attitudes toward dementia, and caregiver burden would be significantly correlated (*p* ≤ .05) with the fear of incompetence.

### 5.2. Discriminant Validity

To ensure that fear of incompetence is distinct from social anxiety and caregiver self-efficacy, discriminant validity was established by assessing the relationship between these two constructs and the FOI-D Scale. First, although fear of incompetence and social anxiety both promote avoidance of face-to-face interactions with other people [7, 19], fear of incompetence is an affective-motivational response to interactional difficulties that specifically arise within the unique relationship with a close family member with dementia. Thus, it is likely that people experiencing greater fear of incompetence do not lack social interactional skills, rather these skills are put to the test in the context of interacting with a close other who has experienced extensive psychological, physical, and personality changes due to dementia. Second, there is evidence that caregiver self-efficacy decreases as the family member's sense of distress and burden increases [32]. However, as conceptualized, fear of incompetence is an affective-motivational response to dementia interactional difficulties as opposed to a cognitive self-assessment of self as an incompetent relational and caregiving partner. Therefore, we expected that the relationship between social anxiety, caregiver self-efficacy, and fear of incompetence would be moderate (i.e., *r* = 0.30) at best.

### 5.3. Concurrent Validity

Because fear of incompetence results in lower desire for interaction with a close family member with dementia [8], it is likely that the quality of one's relationship with the family member with dementia is also negatively affected. Therefore, fear of incompetence was expected to significantly correlate with relationships quality (*p* ≤ .05).

### 5.4. Methods

#### 5.4.1. Participants

A total of 636 North American (218 men, 408 women) adults with a family member with dementia participated in Study Two. The sample was predominately Caucasian (79%), with a mean age of 36.44 years (*SD* = 11.29; range = 18–80 years). Repeat participants from Study 1 (*n* = 47) were not included in this sample. Most of the participants described their family member as their “grandparent” (54%). However, a substantial proportion indicated that their family member was their “parent” (21%), “aunt/uncle” (15%), “great-grandparent” (2%), or “other” (8%). The participants indicated knowing their family member for an average of 34.51 years (*SD* = 12.24; range = 2–73 years) and that their family member was diagnosed with dementia an average of 3.94 years ago (*SD* = 3.36, range = 2 months–30 years).

#### 5.4.2. Measures

 
*Demographic Questionnaire.*

The demographics questionnaire that was used in Study One was adopted in Study Two. However, two item assessing relationship quality and caregiving involvement items were added. With respect to relationship quality, participants were asked to rate their relationship with their family member on an 11-point scale ranging from 0 (very negative) to 11 (very positive). In addition, the caregiving item asked participants to report on the extent are they are involved in providing care for their family member with dementia using an 11-point scale ranging from 1 (not at all involved) to 11 (extremely involved).

 
*The Fear of Incompetence Scale (FOI-D Scale).*

The revised 65-item FOI-D Scale (developed in Study One) was administered in Study Two for CFA purposes.

 
*Dementia Knowledge Scale (DKS).*

The DKS is a 30-item scale that was adapted from Alzheimer's Disease Knowledge Scale by replacing all instances of “Alzheimer's disease” with “Dementia”. These true/false items assessed constructs related to: risk factors, assessment, symptomology, disease progression, life impact, caregiving, and treatment/management. Sample items included: “People with dementia do best with simple instructions giving one step at a time”. and “It has been scientifically proven that mental exercise can prevent a person from getting dementia”. The total DKS score was calculated by summing the correct scores for each item, yielding a total score ranging from 0 to 30. Higher scores indicate more knowledge. The ADKS (the scale from which the DKS was created) has demonstrated adequate content, predictive, concurrent, and convergent validity. In Study Two, the DKS demonstrated adequate internal consistency, *α* = 0.65.

 
*Dementia Attitudes Scale (DAS; [23]).*

The DAS included 20 items that assess the affective, behavioral, and cognitive components of the attitudes toward individuals with dementia. The DAS is organized into two factors “Dementia Knowledge” and “Social Comfort” and includes items such as: “I feel uncomfortable being around people with dementia” and “People with dementia can feel when others are kind to them”. All items on the scale were rated using a 7-point scale ranging from 1 (strongly disagree) to 7 (strongly agree). The DAS has demonstrated excellent psychometric properties [23] and had good internal consistency in Study Two, with a Cronbach's alpha of 0.84.

 
*Burden Scale for Family Caregivers—Short Version (BSFC-S).*

The BSFC-S is a 10-item instrument for assessing self-reported burden among informal caregivers. Sample items included: “My health is affected by the care situation” and “Since I have started providing care, my financial situation has decreased”. Each item is rated on a 4-point scale with the values ranging from 0 (strongly disagree) to 4 (strongly agree). Scale scores were computed by taking the average of all ten items, with higher scores indicating greater burden. The BSFC-S has demonstrated commendable psychometric properties and was internally consistent this study, *α* = 0.87.

 
*Liebewitz Social Anxiety Scale (LSAS).*

The 24-item LSAS assessed fear and avoidance in social interactions in general (11 items) and performance (13 items) in social situations. All items depict social situations that may illicit fear and require that participants rate the extent to which “they experience fear or anxiety in these situations” using a 4-point scale from 0 (never) to 3 (usually). Scale scores were computed by taking the average of all 24 items, with higher scores indicating greater anxiety. The LSAS has repeatedly demstrated excellent psychometric properties and demonstrated great internal consistency in Study Two, *α* = 0.94.

 
*Caregiver Self-Efficacy Scale (CSES).*

The CSES scale was developed to measure caregiver self-efficacy for managing dementia and was comprised of two subscales: symptom management and community support service use. This scale was comprised of 10 items that were assessed using a 9-point scale ranging from 1 (not at all certain) to 10 (very certain). All items began with the phrase: “How certain are you right now that you can….” Sample items included: “deal with the frustrations of caring for your relative” and “find organizations or agencies in the community that provide services to help you care for your relative”. Scale scores were computed by taking the average of all 10 items, with higher scores indicating greater self-efficacy. Unfortunately, little information was provided about the psychometric properties of the CSES. However, the CSES demonstrated excellent internal consistency in this study, *α* = 0.91.

 
*Procedure.*

Participants were recruited on MTurk® using procedures identical to Study One.

### 5.5. Results and Discussion

#### 5.5.1. Confirmatory Factor Analysis

After data cleaning, a CFA using AMOS 16® software was used to replicate and cross-validate the factor structure of the FOI-D Scale identified in Study One. CFA procedures similar to those used in Thompson et al. [33] were adopted for the current study. The results indicated that the proposed CFA model had poor fit (as evidenced by the model fit indices). Following procedures described by Whittaker [34] to improve model fit, overlapping items were identified by examining the modification indices, expected parameter change values, and standardized residual covariances (see our OSF page for final model—https://osf.io/e74v9/?view_only=b8c3f67372dd4f8d82f218d5fd205cee). In addition, model fit indices with and without these items were taken into consideration to determine whether to include these overlapping items in the final scale.

**Figure 1:**
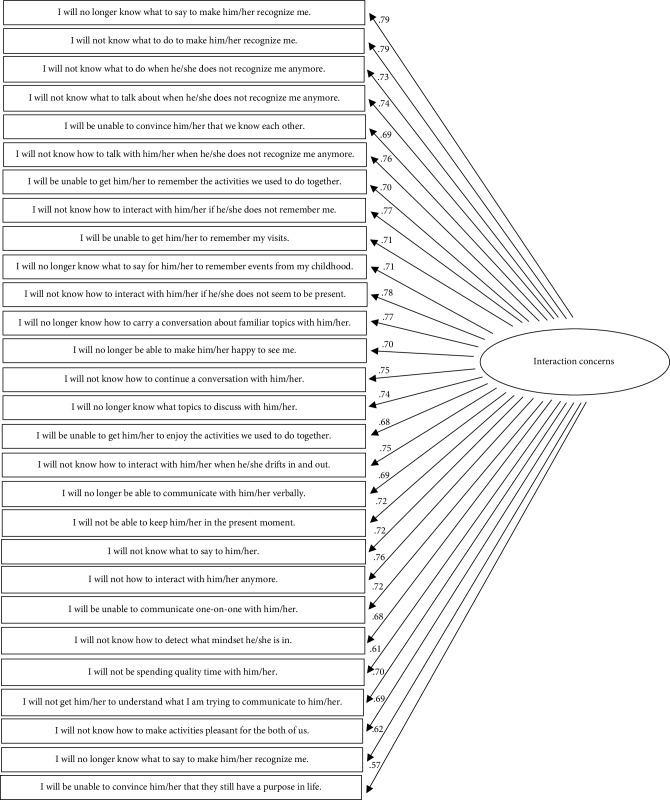
Path diagram depicting the interaction concerns subscale and standardized loadings.

**Figure 2:**
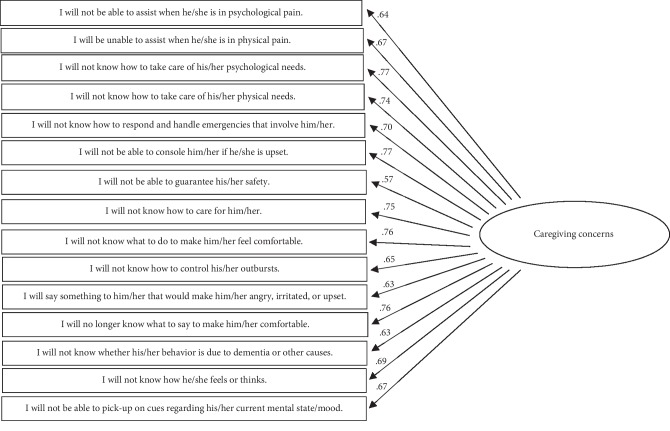
Path diagram depicting the caregiving concerns subscale and standardized loadings.

**Figure 3:**
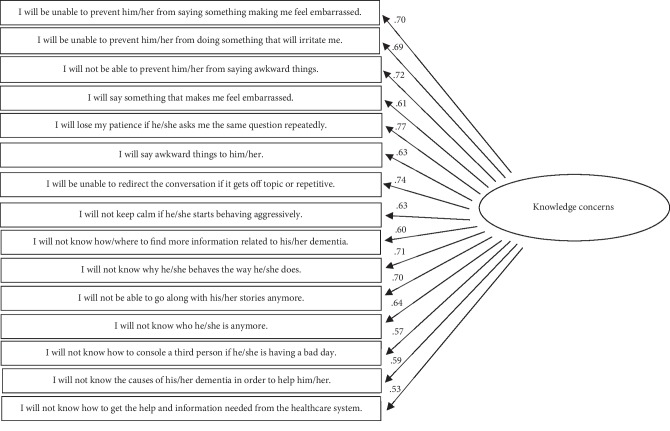
Path diagram depicting the knowledge concerns subscale and standardized loadings. *Note*. The standardized coefficient between the interaction concerns subscale and the caregiving concerns subscale was 0.84.The standardized coefficient between the interaction concerns subscale and the knowledge concerns subscale was 0.77.The standardized coefficient between the caregiving concerns subscale and the knowledge concerns subscale was 0.69.

After removing seven items contributing to poor fit (i.e., overlapping items; see Table 1) and allowing for covariance within factors, a final CFA was conducted (see Figures 1–3 for a visual representation). Although the chi-square statistic is frequently reported as a metric of model fit, this criterion is rarely a useful fit indicator because it is highly sensitive to sample size [35, 36]. Thus, to evaluate model fit the following guidelines were applied: the model must have had a comparative fit index (CFI) approaching .95, a root-mean-square error of approximation (RMSEA) and a standardized root mean squared residual (SRMR) of approximately .06, and a Tucker–Lewis Index (TLI) of approximately .95 [37–39].

All of the model fit indices suggested that the fit of the final FOI-D Scale was adequate. See Table 3 for model fit indices for initial and final model. The factor loadings for all remaining items were significant and performed well. Average standardized factor loadings ranged between 0.49 and 0.79, suggesting good convergent validity among the items in each subscale (see Figures 1–3 for all factor loadings). Thus, the final version of the FOI-D Scale was comprised of 58 items organized into three subscales, with the Interaction Concerns subscale containing 28 items, the Caregiving Concerns subscale that included 15 items, and the Knowledge Concerns subscale comprising 15 items (see Table 4 for descriptive information for each of the final items).

#### 5.5.2. Convergent Validity

As shown in Table 5, only scores on the knowledge subscale of the FOI-D Scale were significantly and negatively correlated with scores on the DKS, indicating that those with greater dementia knowledge reported lower fear of incompetence. Scores on all FOI-D subscales were significantly and negatively related to DAS scores suggesting that those with more positive attitudes toward individuals with dementia reported lower fear of incompetence. Finally, scores on all FOI-D subscales were significantly and positively related to BSFC-S scores. This relationship supports our predictions that adults experiencing greater burden reported greater fear of incompetence. It is worth noting, that although the participants were not required to be caregivers, many indicated average-to-extensive involvement in the care of their family member (as assessed by the caregiving involvement item in the demographics questionnaire). In fact, only 61 of the 636 participants indicated that they were “not at all involved” in caregiving (*M* = 6.61, *SD* = 3.25, rated on an 11-point scale). However, despite the emergence of a caregiving subscale, the extent to which participants provided care to their family member was not significantly correlated with any of the FOI subscales (*r*_interaction_ = 0.08, *r*_caregiving_ = 0.04; *r*_knowledge_ = 0.03; *p*s > .05). These results indicate that although caregiving is a source of fear of incompetence, serving as a caregiver is not a prerequisite for experiencing these fears.

#### 5.5.3. Discriminant Validity

LSAS scores were only moderately correlated with scores on all FOI-D subscales. This suggests that, although related, social anxiety is distinct from fear of incompetence. In addition, the correlations between all FOI-D subscales were slightly correlated at best with CSES scores, suggesting that self-efficacy and fear of incompetence are distinct constructs (see Table 5). Although these constructs met the criteria necessary for establishing discriminant validity, it is important to note that the relationship between all constructs were still statistically significant. These findings indicate that, despite being distinct, fear of incompetence is a broad measure and has implications for one's self-efficacy and anxiety when interacting with a family member with dementia.

#### 5.5.4. Concurrent Validity

Three bivariate correlations were conducted in which the association between scores on all three FOI-D subscales and the relationship quality variable were assessed. The results revealed that only the Interaction Concerns subscale was significantly and negatively correlated with relationship satisfaction (*r* = −0.11, *p* = .01), suggesting that those with greater interactional fear of incompetence reported lower relationship satisfaction.

#### 5.5.5. Internal Consistency

All three subscales demonstrated excellent consistency based on Cronbach's alphas (Interaction Concerns subscale* *= 0.96; Caregiving Concerns subscale* *= 0.90; Knowledge Concerns subscale* *= 0.90). In sum, the results from Study Two provide evidence of the validity of the factor structure as well as the adequate psychometric properties of the FOI-D Scale.

## 6. Study Three: Temporal Consistency

To establish temporal consistency of the FOI-D Scale, a subset of participants from Study Two completed the scale for a second time, approximately ten weeks after the initial administration. It was expected that all subscales of the FOI-D Scale would demonstrate adequate test-retest reliability as evidenced by an intra-class correlating coefficients (ICCs) of 0.75 or more. The internal consistency of each subscale was assessed again in order to ensure adequate scale reliability.

### 6.1. Method

#### 6.1.1. Participants

One hundred of the participants from Study Two were e-mailed a link to participate in Study Three. Of the 100 who were e-mailed the survey link, 58 (18 men, 40 women) participated for a second time. These participants were predominately Caucasian (78%), with a mean age of 37.76 years (*SD *= 12.49; range* *= 22–72 years). Most of the participants described their family member as their “grandparent” (42%). The rest indicated that their family member was their “parent” (32%), “aunt/uncle” (14%), “great-grandparent” (3%), or some other type of relationship (9%). The participants indicated knowing their family member for an average of 34.70 years (*SD *= 13.73; range* *= 3–67 years) and that their family member was diagnosed with dementia an average of 4.19 years ago (*SD *= 4.05, range* *= 6 months–30 years).

#### 6.1.2. Measures

The measures for Study Three included the demographics questionnaire and the final FOI-D Scale from Study Two.

#### 6.1.3. Procedure

All participants were sent an e-mail with a link to participate in Study Three. Then, four reminders were distributed within a two-week period. After two weeks, the study was closed. Interested participants were directed to the consent form and then the online survey (which took 20 minutes to complete). Afterward, participants were presented with the online debriefing form and received $0.50 USD for their time.

### 6.2. Results and Discussion

Independent *t*-tests were conducted to examine group differences between those who participated in the follow-up study and those who did not. Participants completing Study Three did not differ significantly from those who did not, in terms of their FOI-D Scale scores, gender, or age (all *p*s > .05). Descriptive statistics revealed that participants reported moderate levels of fear of incompetence as evidenced by means on the three subscales ranging from 2.69 to 3.13 on the 5-point scale. The average score on the Interaction Concerns subscale was 3.13 (*SD *= 0.86), the Caregiving Concerns subscale was 3.28 (*SD *= 0.86), and the Knowledge Concerns subscale was 2.69 (*SD *= 0.89).

The Cronbach alphas indicated that all three subscales demonstrated adequate scale reliability (Interaction Concerns subscale* *= 0.96; Caregiving Concerns subscale* *= 0.92; Knowledge Concerns subscale* *= 0.89). In addition, the results from ICCs revealed acceptable ten-week temporal consistency for the FOI-D Scale, as evidenced by a strong positive intraclass correlation between the first and second administration, *r* (57)* *= 0.86, *p* < .001. When examining each subscale independently, the Interaction Concerns subscale had a correlation of 0.91 (*p* < .001), the Caregiving Concerns subscale had a correlation of 0.88 (*p* < .001), and the Knowledge Concerns subscale had a correlation of 0.90 (*p* < .001). Overall, Study Three results indicated stability across time.

## 7. General Discussion

The primary objective of the current program of research was to investigate fear of incompetence among adults with a family member diagnosed with dementia by developing, refining, and psychometrically validating a comprehensive measure assessing the fear of incompetence. The FOI-D scale measured a variety of interactional concerns that were organized into three factors: interaction, caregiving, and knowledge concerns. The scale demonstrated excellent internal consistency (Studies 1–3) and psychometric properties (Studies 2-3).

The final FOI-D Scale measured all interactional concerns documented in Miron and colleagues' work [9] as well as other previous studies (e.g., [23]). In fact, concerns about lacking perspective-taking, self-regulation skills, conversation, and interaction skills all fell under the Interaction Concerns subscale, concerns about lacking knowledge about dementia under the Knowledge Concerns subscale, and concerns about one's caregiving skills/knowledge under the Caregiving Concerns subscale.

### 7.1. Theoretical, Clinical, and Practical Implications

In the current program of research, fear of incompetence was clearly experienced by most adults with a loved one with dementia as evidenced by the range of scores obtained from the FOI-D, which speaks to the importance of assessing fear of incompetence when studying interactions with family members with dementia. The need to understand people's experience with fear of incompetence is becoming even more important as the number of individuals with dementia and the number of family caregivers are expected to increase [40, 41].

In the future, researchers could examine the various outcomes of fear of incompetence, including family members' decision to place their loved one with dementia in institutionalized care or the amount of time spent providing care for the person. Family caregivers' decisions to transition a family member with dementia from home care to institutionalized care can be conceptualized as a more extreme form of avoidance of face-to-face interaction with the person with dementia. Face-to-face interactions allow for more meaningful interaction and relationship experiences [9, 18, 19]. Moreover, repeated moves between care settings as well as permanent transitions of persons with dementia from home care to institutionalized care come with a host of negative outcomes for the person with dementia and their family caregivers [42–44]. Thus, it is important to assess the role of fear of incompetence in motivating care transition decisions by family caregivers and develop strategies to reduce it as a means of curtailing these care transitions.

Relatedly, the results of the current program of research also have implications for family members providing care. By differentiating fear of incompetence from trait social anxiety and expanding the notion of caregiver self-efficacy (or lack thereof), interventions designed to improve interactions can now be more easily conceived that target family members. Although the family members in the current research were not required to have assumed a caregiving role, nearly all participants indicated providing care for their family member in some capacity. This likely explains why caregiving emerged as one of the three components of fear of incompetence in interactions with loved ones with dementia. The caregiving component of fear of incompetence among family members of adults diagnosed with dementia is also consistent with research indicating that noncaregivers are routinely sought out for physical, emotional, social, or financial assistance [45]. Therefore, interventions looking to reduce fear of incompetence (e.g., communication skills education) should not exclusively target those in traditional, formal, caregiving roles.

Another implication stemming from the current program of research relates to the importance of communication. In fact, the results reported here suggest that person-centered approaches that focus on improving communication with diagnosed relatives are warranted because they could reduce fear of interactional incompetence in family members of persons with dementia. For example, Mittelman et al. [14] found that counseling sessions and access to support groups reduced the frequency of spouses being admitted to a nursing home following a diagnosis of Alzheimer disease. Person-centered trainings focusing on the development and improvement of communication skills among individuals providing dementia care have proven to be effective and result in significant improvements to the quality of life and wellbeing of people with dementia (see [5] for review). However, most of these trainings are designed for professional caregivers. Thus, greater effort is needed in order to provide these person-centered trainings to family members to improve familial communication and ultimately wellbeing of people with dementia, such as family workshops described by Shulman and Mandel [17] on how to effectively use structured questions when communication is difficult. Future research should focus on directly testing the effectiveness of workshops targeting the interaction, communication, and caregiving skills of those who have family members with dementia in reducing each of the three components of fear of incompetence we documented in the current work.

Finally, broadly speaking, although the FOI-D Scale was developed in an attempt to measure fear of incompetence among adults interacting with family members with dementia, it is likely that fear of interactional incompetence is experienced in other contexts, such as during interactions that occur with individuals who have been diagnosed with other disorders (e.g., Post-Traumatic Stress Disorder, traumatic brain injuries). Furthermore, most family members are motivated to maintain their relationship regardless of the type of disorder their loved one is experiencing. Thus, not only is the FOI-D Scale a valuable tool for understanding interactions occurring with individuals with dementia, it likely will also prove useful for researchers, educators, and practitioners assessing fear of incompetence in a variety of contexts.

### 7.2. Limitations and Future Directions

Several limitations of the current research must be noted. First, the samples obtained in the current program of research were comprised of the U.S. residents who were predominately young Caucasian women. As a result, the current findings may not characterize the concerns present among individuals in nonwestern cultures. Therefore, studies adopting a more expansive and inclusive recruitment methodology to assess fear of incompetence are needed.

Second, the FOI-D was self-report in design and although all data were collected online and participants were assured that anonymity would be maintained, demand characteristics and socially desirable responding may still have influenced participants' responses (particularly when reporting fears that are socially sensitive in nature). Thus, future research should employ nontraditional measures (e.g. the implicit association test, bogus pipeline procedures) or include measures of social desirability to overcome some of these responses biases or help control for their effects.

Third, the FOI-D only accounted for a total of 51.75% of the variance, which is lower than the suggested cut-off of 60% proposed by Hinkin [46]. This suggests that random error is present in our measure and may limit the validity of the measure. This error might be a result of variability associated with what type of support adults provide their family members, their frequency of contact, their co-residence status, and the nature of their relationship with the family member (e.g., child, grandchild, niece/nephew). Thus, future research should attempt to replicate these results by using different samples and assessing potential covariates in order to investigate the source of some of the unexplained variation.

Finally, some of the measures used to establish the psychometric properties of the scale could be improved. For example, relationship quality was only assessed using a single item with limited internal validity. In addition, trait anxiety could have been assessed in addition to social anxiety. Consequently, researchers should continue testing the validity the FOI-D using additional measures and constructs.

## 8. Conclusions

Overall, as the prevalence of dementia continues to rise and the care of individuals with dementia continues to shift toward family members, there is a growing need to assess fear of interactional incompetence. Thus, because of its strong psychometric properties, the FOI-D scale can offer a variety of applications for future investigators, educators, and practitioners interested in improving interpersonal interactions and relationships.

## Figures and Tables

**Table 1:** Items omitted from the FOI scale at each stage.

Items omitted
*EFA*
I will be unable to redirect him/her when he/she does not know where they are.
I will not know what to expect (e.g., what mood he/she is in when you enter the room).
I will not know when and how to touch him/her (e.g., hug him/her when we first meet up).
I will not know when and how to touch him/her (e.g., hug him/her when we first meet up).
I will not know what reality he/she is currently in when trying to interact.
I will not know how to react if he/she behaves unpredictably.
I will not know how to deal with his/her mood swings.
I will not understand what he/she is trying to say to me.
I will be unable to redirect him/her when he/she wants to go home.
I will say something that will confuse him/her.
I will not know whether he/she likes to be touched or not (e.g., hug him/her when we first meet up).
I will not know what conversation topics bring back bad memories.
I will no longer know what he/she likes or does not like.
I will not be spending enough time with him/her.
I will say the wrong thing to him/her.
I will not know how to find out about and set up services for him/her.
I will not know what to say if I visit him/her alone, without a relative or friend.
I will not know what to do if I were to visit him/her by myself, without a relative or friend.
My interactions with him/her will be awkward.
I will not know enough about dementia to help in a meaningful way.
I will no longer be able to follow our regular routine.
I will not know how to assist a third person in the interaction (e.g., sibling, parent, friend) if my relative with dementia does not recognize them.

*CFA*
I will do something that will irritate him/her.
I will not know how to calm him/her down.
I will not know what to do to make him/her remember events from my childhood.
I will not know the causes of his/her dementia in order to prevent it from happening to me.
I will not have enough knowledge about dementia to communicate effectively.
I will not be able to detect the amount of physical pain he/she is in.
I will not be able to detect the amount of psychological pain he/she is in.

*Note.* Scale instructions read as follows: “Below are several concerns that people may have when interacting with their relative or family member who has been diagnosed with dementia. Using the rating scale provided, please indicate to what extent each of the following would concern you when interacting with a living family member with dementia.”

**Table 2:** Factor loadings for the items in the FOI scale based on the final maximum likelihood exploratory factor analysis with a promax rotation.

FOI scale items				
Scale instructions read as follows: “Below are several concerns that people may have when interacting with their relative or family member who has been diagnosed with dementia. Using the rating scale provided, please indicate to what extent each of the following would concern you when interacting with a living family member with dementia.”	1	2	3	*h* ^2^

I will not know what to talk about when he/she does not recognize me anymore.	**.93**	−.13	−.07	.64
I will not know what to do when he/she does not recognize me anymore.	**.91**	.03	−.25	.64
I will not know how to talk with him/her when he/she does not recognize me anymore.	**.91**	−.01	−.16	.64
I will not know what to do to make him/her recognize me.	**.88**	.01	−.11	.68
I will not know how to interact with him/her if he/she does not remember me.	**.87**	−.01	−.13	.61
I will be unable to convince him/her that we know each other.	**.82**	.04	−.15	.59
I will no longer know what to say to make him/her recognize me.	**.79**	.03	−.09	.58
I will be unable to get him/her to remember the activities we used to do together.	**.78**	−.10	.05	.59
I will be unable to get him/her to remember my visits.	**.72**	−.12	.13	.57
I will no longer know what to say for him/her to remember events from my childhood.	**.70**	−.11	.11	.53
I will no longer know how to carry a conversation about familiar topics with him/her.	**.67**	−.00	.12	.58
I will be unable to get him/her to enjoy the activities we used to do together.	**.64**	.03	.06	.52
I will not know how to continue a conversation with him/her.	.63	−.08	.24	.58
I will no longer be able to make him/her happy to see me.	**.63**	.16	−.10	.50
I will not know what to do to make him/her remember events from my childhood.	**.63**	−.10	.21	.55
I will not know what to say to him/her.	**.61**	−.04	.24	.57
I will not know how to interact with him/her when he/she drifts in and out.	**.60**	.08	.14	.61
I will no longer know what topics to discuss with him/her.	**.59**	−.00	.18	.53
I will not how to interact with him/her anymore.	**.59**	.13	.10	.60
I will no longer be able to communicate with him/her verbally.	**.54**	.18	.01	.49
I will not be able to keep him/her in the present moment.	**.52**	.11	.11	.49
I will not know how to interact with him/her if he/she does not seem to be present.	**.52**	.18	.10	.54
I will be unable to communicate one-on-one with him/her.	**.49**	.28	.05	.50
I will not get him/her to understand what I am trying to communicate to him/her.	**.48**	.27	−.03	.48
I will no longer know what to say to make him/her comfortable.	**.46**	.37	−.02	.58
I will no longer be able to prevent him/her from rejecting me.	**.43**	−.00	.25	.42
I will not know how to detect what mindset he/she is in (e.g., recent times or memories from his/her past)	**.42**	.33	.05	.54
I will not be spending quality time with him/her.	**.42**	.09	.11	.34
I will not know how to make activities pleasant for the both of us.	**.40**	.25	.13	.49
I will be unable to assist when he/she is in physical pain.	−.17	**.89**	−.12	.57
I will not be able to detect the amount of physical pain he/she is in.	−.09	**.88**	−.16	.57
I will not be able to detect the amount of psychological pain he/she is in.	.02	**.85**	−.21	.59
I will be unable to assist when he/she is in psychological pain.	.11	**.78**	−.22	.57
I will not be able to guarantee his/her safety.	−.14	**.77**	−.03	.48
I will not know how to take care of his/her psychological needs.	.12	**.77**	−.17	.59
I will not know how to take care of his/her physical needs.	−.19	**.76**	.14	.56
I will not know what to do to make him/her feel comfortable.	.27	**.66**	−.14	.63
I will not know how to care for him/her.	−.01	**.65**	.07	.53
I will not be able to console him/her if he/she is upset.	.26	.63	−.19	.52
I will not know how to calm him/her down.	.21	**.60**	−.07	.53
I will not know how to respond and handle emergencies that involve him/her.	−.09	**.59**	.19	.46
I will say something to him/her that would make him/her angry, irritated, or upset.	.07	**.53**	.12	.45
I will do something that will irritate him/her.	−.03	**.49**	.21	.40
I will not be able to pick-up on cues regarding his/her current mental state/mood.	.12	**.46**	.18	.47
I will not know whether a behavior displayed by him/her is due to dementia or other causes.	.06	**.45**	.21	.45
I will not know how he/she feels or thinks.	.36	**.44**	−.05	.51
I will be unable to convince him/her that they still have a purpose in life.	.22	**.42**	.04	.40
I will not know how to control his/her outbursts.	.09	**.41**	.23	.44
I will be unable to prevent him/her from saying something that makes me feel embarrassed.	−.06	−.19	**.81**	.52
I will be unable to prevent him/her from doing something that will irritate me.	−.06	−.12	**.79**	.55
I will say something that makes me feel embarrassed.	.00	−.14	**.76**	.51
I will not be able to prevent him/her from saying awkward things.	.11	−.20	**.73**	.53
I will lose my patience if he/she asks me the same question repeatedly.	.02	−.12	**.69**	.48
I will not know how/where to find more information related to his/her dementia.	−.19	.17	**.67**	.45
I will not be able to go along with his/her stories anymore.	.23	−.17	**.64**	.53
I will not keep calm if he/she starts behaving aggressively.	−.09	.10	**.63**	.46
I will say awkward things to him/her.	.00	.06	**.60**	.43
I will not know how to get the help and information needed from the healthcare system.	−.27	.39	**.54**	.45
I will be unable to redirect the conversation if it gets off topic or repetitive.	.29	−.11	**.54**	.52
I will not have enough knowledge about dementia to communicate effectively.	−.09	.32	**.51**	.48
I will not know why he/she behaves the way he/she does.	.13	.09	**.48**	.45
I will not know the causes of his/her dementia in order to help him/her.	−.02	.22	**.47**	.41
I will not know the causes of his/her dementia in order to prevent it from happening to me.	.01	.18	**.42**	.35
I will not know who he/she is anymore.	.24	.00	**.42**	.41
I will not know how to console a third person in the interaction (e.g., sibling, parent, friend) if my relative with dementia is having a bad day.^∗^	.13	.21	**.36**	.40

*Note.* 1 = Interaction Concerns subscale, 2 = Caregiving Concerns subscale, 3 = Knowledge Concerns subscale. Participants were provided the following instructions “Below are several concerns that people may have when interacting with their relative or family member who has been diagnosed with dementia. Using the rating scale from 1 (not at all concerned) to 7 (extremely concerned), please indicate to what extent each of the following would concern you when interacting with a living family member with dementia.” ^∗^ = item retained at the researcher's discretion.

**Table 3:** Confirmatory factor analysis model fit indices.

	RMSEA	SRMR	CFI	TLI
Initial model	0.07	0.08	0.90	0.88
Final model	0.05	0.07	0.91	0.91

*Note*. Comparative Fit Index = CFI, Root-Mean-Square Error of Approximation  = RMSEA, Standardized Root Mean Squared Residual = SRMR, Tucker–Lewis Index = TLI.

**Table 4:** Means and standard deviations for the items in the final FOI scale.

FOI scale items	*Mean*	*SD*
Scale instructions read as follows: “Below are several concerns that people may have when interacting with their relative or family member who has been diagnosed with dementia. Using the rating scale provided, please indicate to what extent each of the following would concern you when interacting with a living family member with dementia.”		

*Interaction concerns subscale*		
I will not know what to do when he/she does not recognize me anymore.	3.52	1.23
I will be unable to convince him/her that we know each other.	3.46	1.24
I will no longer be able to communicate with him/her verbally.	3.42	1.26
I will no longer know what to say to make him/her recognize me.	3.39	1.21
I will not know how to talk with him/her when he/she does not recognize me anymore.	3.39	1.27
I will no longer know what to say to make him/her comfortable.	3.38	1.18
I will not get him/her to understand what I am trying to communicate to him/her.	3.37	1.15
I will not know what to do to make him/her recognize me.	3.35	1.22
I will not know how to interact with him/her if he/she does not remember me.	3.34	1.24
I will be unable to communicate one-on-one with him/her.	3.31	1.23
I will no longer be able to make him/her happy to see me.	3.31	1.20
I will not know how to detect what mindset he/she is in (e.g., recent times or memories from his/her past).	3.31	1.12
I will not know what to talk about when he/she does not recognize me anymore.	3.30	1.23
I will be unable to get him/her to enjoy the activities we used to do together.	3.30	1.20
I will not how to interact with him/her anymore.	3.27	1.23
I will not know how to interact with him/her when he/she drifts in and out.	3.21	1.17
I will be unable to get him/her to remember the activities we used to do together.	3.20	1.19
I will not get him/her to understand what I am trying to communicate to him/her.	3.18	1.23
I will not be able to keep him/her in the present moment.	3.18	1.23
I will not know how to interact with him/her if he/she does not seem to be present.	3.16	1.23
I will not know how to continue a conversation with him/her.	3.14	1.22
I will be unable to get him/her to remember my visits.	3.14	1.23
I will not know how to make activities pleasant for the both of us.	3.07	1.19
I will not be spending quality time with him/her.	3.07	1.30
I will not know what to say to him/her.	3.07	1.25
I will no longer know what topics to discuss with him/her.	3.07	1.23
I will no longer know what to say for him/her to remember events from my childhood.	3.03	1.23
I will no longer be able to prevent him/her from rejecting me.	2.95	1.29

*Caregiving concerns scale*		
I will be unable to assist when he/she is in psychological pain.	3.62	1.19
I will not know how to take care of his/her psychological needs.	3.59	1.17
I will not be able to console him/her if he/she is upset.	3.56	1.20
I will not know how he/she feels or thinks.	3.51	1.15
I will be unable to assist when he/she is in physical pain.	3.49	1.22
I will not know what to do to make him/her feel comfortable.	3.49	1.16
I will not know how to control his/her outbursts.	3.42	1.22
I will not be able to guarantee his/her safety.	3.40	1.33
I will not know how to take care of his/her physical needs.	3.38	1.23
I will say something to him/her that would make him/her angry, irritated, or upset.	3.35	1.18
I will not know how to care for him/her.	3.35	1.25
I will not know whether a behavior displayed by him/her is due to dementia or other causes.	3.34	1.21
I will not know how to respond and handle emergencies that involve him/her.	3.34	1.25
I will be unable to convince him/her that they still have a purpose in life.	3.31	1.25
I will not be able to pick-up on cues regarding his/her current mental state/mood.	3.17	1.73

*Knowledge concerns subscale*		
I will not know the causes of his/her dementia in order to help him/her.	3.09	1.27
I will not know how to console a third person in the interaction (e.g., sibling, parent, friend) if my relative with dementia is having a bad day.	3.09	1.23
I will not know how to get the help and information needed from the healthcare system.	2.86	1.27
I will not know why he/she behaves the way he/she does.	2.85	1.23
I will not know who he/she is anymore.	2.78	1.37
I will be unable to redirect the conversation if it gets off topic or repetitive.	2.75	1.24
I will not keep calm if he/she starts behaving aggressively.	2.73	1.36
I will not be able to go along with his/her stories anymore.	2.68	1.24
I will not be able to prevent him/her from saying awkward things.	2.57	1.29
I will lose my patience if he/she asks me the same question repeatedly.	2.54	1.37
I will be unable to prevent him/her from doing something that will irritate me.	2.49	1.27
I will not know how/where to find more information related to his/her dementia.	2.49	1.29
I will be unable to prevent him/her from saying something that makes me feel embarrassed.	2.46	1.22
I will say awkward things to him/her.	2.32	1.22
I will say something that makes me feel embarrassed.	2.15	1.22

*Note*. All items rated on a 5-point scale from 1 (not at all concerned) to 5 (extremely concerned).

**Table 5:** Correlation coefficients and descriptive statistics for the FOI subscales and additional measures.

Study variables	Pearson-product moment correlation coefficients							
	ICS	CCS	KCS	DKS	DAS	BSFC-S	LSAS	CSES
ICS		.81^∗∗∗^	.77^∗∗∗^	−.05	−.13^∗∗^	−.16^∗∗^	.23^∗∗∗^	−.07
CCS			.68^∗∗∗^	.04	−.09^∗^	−.16^∗∗^	.27^∗∗∗^	−.13^∗∗^
KCS				−.20^∗∗∗^	−.30^∗∗∗^	−.18^∗∗^	.27^∗∗∗^	−.07
*Mean* (*SD*)	3.35 (0.84)	3.36 (0.85)	2.63 (0.89)	5.29 (0.12)	4.82 (0.75)	2.35 (0.61)	2.21 (0.65)	5.96 (1.81)

*Note.* ^∗∗∗^ **= *p* < .001, ^∗∗^* = p* < .01, ^∗^* *= *p* < .05. ICS* *= Interaction Concerns Subscale, CCS* *= Caregiving Concerns Subscale, KCS* *= Knowledge Concerns Subscale, DKS* *= Dementia Knowledge Scale, DAS* *= Dementia Attitudes Scale, BSFC-S* *= Burden Scale for Family Caregivers—Short Version, LSAS* *= Liebewitz Social Anxiety Scale, CSES* *= Caregiver Self-Efficacy Scale.

## Data Availability

As part of the *International Journal of Alzheimer's Disease's* encouragement of open research practices, the authors have made the data used in the research available at: https://osf.io/e74v9/. The materials used in the research can also be made available by emailing: thompsoa@d.umn.edu.
